# Inter-database validation of a deep learning approach for automatic sleep scoring

**DOI:** 10.1371/journal.pone.0256111

**Published:** 2021-08-16

**Authors:** Diego Alvarez-Estevez, Roselyne M. Rijsman

**Affiliations:** 1 Sleep Center, Haaglanden Medisch Centrum, The Hague, South-Holland, The Netherlands; 2 Center for Information and Communications Technology Research (CITIC), University of A Coruña, A Coruña, Spain; University of Oklahoma, UNITED STATES

## Abstract

**Study objectives:**

Development of inter-database generalizable sleep staging algorithms represents a challenge due to increased data variability across different datasets. Sharing data between different centers is also a problem due to potential restrictions due to patient privacy protection. In this work, we describe a new deep learning approach for automatic sleep staging, and address its generalization capabilities on a wide range of public sleep staging databases. We also examine the suitability of a novel approach that uses an ensemble of individual local models and evaluate its impact on the resulting inter-database generalization performance.

**Methods:**

A general deep learning network architecture for automatic sleep staging is presented. Different preprocessing and architectural variant options are tested. The resulting prediction capabilities are evaluated and compared on a heterogeneous collection of six public sleep staging datasets. Validation is carried out in the context of independent local and external dataset generalization scenarios.

**Results:**

Best results were achieved using the CNN_LSTM_5 neural network variant. Average prediction capabilities on independent local testing sets achieved 0.80 kappa score. When individual local models predict data from external datasets, average kappa score decreases to 0.54. Using the proposed ensemble-based approach, average kappa performance on the external dataset prediction scenario increases to 0.62. To our knowledge this is the largest study by the number of datasets so far on validating the generalization capabilities of an automatic sleep staging algorithm using external databases.

**Conclusions:**

Validation results show good general performance of our method, as compared with the expected levels of human agreement, as well as to state-of-the-art automatic sleep staging methods. The proposed ensemble-based approach enables flexible and scalable design, allowing dynamic integration of local models into the final ensemble, preserving data locality, and increasing generalization capabilities of the resulting system at the same time.

## Introduction

Sleep staging is one of the most important tasks during the clinical examination of polysomnographic sleep recordings (PSGs). A PSG records the relevant biomedical signals of a patient in the context of Sleep Medicine studies, representing the basic tool for the diagnosis of many sleep disorders. Sleep staging characterizes the patient’s sleep macrostructure leading to the so-called hypnogram. The hypnogram plays also a fundamental role for the interpretation of several other biosignal activities of interest, such as the evaluation of the respiratory function, or the identification of different body and limb movement [1,2]. Current standard guidelines for sleep scoring carry out segmentation of the subject’s neurophysiological activity following a discrete 30s-epoch time basis. Each epoch can be classified into five possible states (wakefulness, stages N1, N2, N3, and R) according to the observed signal pattern activity in the reference PSG interval. Specifically, for sleep staging, neurophysiological activity of interest involves monitoring of different traces of electroencephalographic (EEG), electromyographic (EMG) and electrooculographic (EOG) activity [1].

A typical PSG examination comprises 8 up to 24 hours of continuous signal recording, and its analysis is usually carried out manually by an expert clinician. The scoring process is consequently expensive and highly demanding, due to the involved clinician’s time, and the complexity of the analysis itself. Moreover, the demand for PSG investigations is growing in relation with the general public awareness, motivated by clinical findings over the last years uncovering the negative impact that sleep disorders exert over health. This represents a challenge for the already congested sleep centers, with steadily increasing waiting lists.

Automatic analysis of the sleep macrostructure is thus of interest, given the potential great savings in terms of time and human resources. An additional advantage is the possibility of providing deterministic (repeatable) diagnostic outcomes, hence contributing to the standardization and quality improvement in the diagnosis. The topic, in fact, is not new, and first related approximations can be traced back to the 1970’s [3,4]. Numerous attempts have followed since then and up to now [5–14], evidencing that the task still represents a challenge, and an open area of research interest. More recently, several approximations have been appearing based on the use of deep learning, claiming advantages over previous realizations which include improved performance, and the possibility to skip handcrafted feature engineering processes [15–23]. However, despite the promising results reported in some of these works, practical acceptance of these systems among the clinical community remains low. Effectively, an unsolved problem remains the inability of these systems to sustain their results beyond the research lab, failing to make them extensible to the practical clinical environment. The problem is closely related with the so-called *database variability problem*, whereby the automatic scoring algorithm is not able to hold its performance beyond a specific testing dataset or the original experimental conditions. More specifically, estimation of the algorithm´s performance is commonly approached using a subset of independent (testing) data, taken from the whole set available in a specific reference database. This testing subset, while independent of the training data, remains effectively “local” to the reference database, meaning training and testing data share characteristics bounded to their common data generation process. However, when considering a multiple-database validation scenario, heterogeneity associated with the various external data sources adds an extra component of variability. In the case of sleep staging, sources of data variability are multiple and include, for example, differences among the subject’s conditions or physiology, the signal acquisition and digitalization methods (e.g. sampling rates, electrode positions, amplification factors or noise-to-signal ratios), and also important, disagreement among expert’s interpretation due to the inherent human subjectivity, or different training backgrounds. Detailed discussion on the topic can be found in a previous work of the authors [24], in which a general trend of performance degradation has been reported among the few works that have attempted validation procedures involving multiple independent external databases.

In this work we describe a new deep learning approach for automatic sleep staging. Given the scarcity of comprehensive validation studies in the literature, one of the major contributions of this work involves addressing the real generalization capabilities of the learning model on a wide range of public sleep staging databases. For this purpose, prediction performance of the proposed approach is evaluated, for each database, in the context of both, independent local, and external generalization scenarios. In the first case, part of each dataset is set aside to be used as independent testing set, while the rest of the data are used for training and parameterization of the machine learning model. On the second scenario (external database validation) the whole dataset is presented as *brand-new* to the model, which was derived based on data from external and completely independent database(s). Effectively, by comparing both procedures it is possible to extrapolate the expected performance of the method, regardless of a specific local database used for the development of the model; hence, a better estimation of the real generalization capabilities of the algorithm on the general reference task of sleep staging can be achieved. Architecture of the proposed deep learning approach uses a novel flexible design combining different layers of Convolutional Neural Networks (CNN) and Long-Short Term Memory (LSTM). The new design adds the capacity for learning of transition rules among sleep stages, i.e. epoch sequence learning, resulting on improved performance of the approach. We investigate the use of different architectural variants and epoch sequence lengths to analyze their impact on the generalization of the resulting models. In addition, we also examine the suitability of a novel approach introduced on a previous work [24], based on the use of an ensemble of individual local models. This approach has potential advantages in terms of modelling and learning scalability, and at the same time, it reduces the necessity of exchanging data between centers for the development of generalizable machine learning models. The impact of this novel ensemble approach on the resulting inter-database generalization performance is also evaluated using the new deep learning approach introduced in this work. Validation results are contextualized with respect to the expected levels of human agreement, and the performance of current state-of-the-art automatic scoring solutions on the sleep staging task. Our approach shows robust behavior in comparison with the available references.

## Materials and methods

### Datasets

A set of heterogeneous and independent clinical sleep scoring datasets was used as testing benchmark during the course of our experiments. In order to enhance reproducibility, all datasets were gathered from public online repositories, and recordings were digitally encoded using the open EDF(+) format [25,26]. An overview of the general characteristics of each integrating dataset is given next. Extended description, including specifications of the corresponding signal montages can be found in S1 Table.

#### Haaglanden Medisch Centrum Sleep Center Database (HMC)

This dataset includes a total of 154 PSG recordings gathered retrospectively from the sleep center database of the Haaglanden Medisch Centrum (The Netherlands). Recordings were randomly selected from a heterogeneous population which was referred for PSG examination on the context of different sleep disorders during the year 2018. Data were acquired in the course of common clinical practice, and thus did not subject people to any other treatment nor prescribed any additional behavior outside of the usual clinical procedures. PSGs were anonymized avoiding any possibility of individual patient identification. Explicit participant consent was not required by the ethics committee due to the retrospective nature of the study and the fact that data were de-identified. Study was approved under identification code METC-19-065. The dataset has been made publicly available online [27].

#### St. Vicent’s Hospital/University College Dublin Sleep Apnea Database (Dublin)

This dataset contains 25 full overnight PSGs from adult subjects with suspected sleep-disordered breathing. Subjects were originally randomly selected over a 6-month period (September 02 to February 03) from patients referred to the Sleep Disorders Clinic at St Vincent’s University Hospital, Dublin, for possible diagnosis of obstructive sleep apnea, central sleep apnea or primary snoring. The 2011 revised version of the dataset was used which is available online on the PhysioNet website [28].

#### Sleep Health Heart Study (SHHS)

The Sleep Heart Health Study (SHHS) is a multi-center cohort study implemented by the National Heart Lung & Blood Institute to determine the cardiovascular and other consequences of sleep-disordered breathing. The database is available online upon permission at the National Sleep Research Resource (NSRR) [29,30]. More information about the rationale, design, and protocol of the SHHS study can be found in the dedicated NSRR section [30] and in the literature [31,32]. For this study a random subset of 100 PSG recordings were selected from the SHHS-2 study. A list of the recording numbers included in the selection is included as supplementary information for reproducibility purposes (S1 File).

#### Sleep Telemetry Study (Telemetry)

This dataset contains 44 whole-night PSGs obtained in a 1994 study of temazepam effects on sleep in 22 caucasian males and females without other medication. Subjects had mild difficulty falling asleep but were otherwise healthy. The PSGs were recorded in the hospital during two nights, one of which was after temazepam intake, and the other of which was after placebo intake. More details on the subjects and the recording conditions are further described in the works of Kemp et al. [33,34]. The dataset is fully available at the PhysioNet website as part of the more extensive Sleep-EDF database [35].

#### DREAMS subject database (DREAMS)

The DREAMS dataset is composed of 20 whole-night PSG recordings from healthy subjects. It was collected during the DREAMS project, to tune, train, and test automatic sleep staging algorithms [36]. The dataset is available online granted by University of MONS—TCTS Laboratory (Stéphanie Devuyst, Thierry Dutoit) and Université Libre de Bruxelles—CHU de Charleroi Sleep Laboratory (Myriam Kerkhofs) under terms of the Attribution-NonCommercial-NoDerivs 3.0 Unported (CC BY-NC-ND 3.0) [37].

#### ISRUC-SLEEP dataset (ISRUC)

This dataset is composed of 100 PSGs from adult subjects with evidence of having sleep disorders. PSG recordings were originally selected from the Sleep Medicine Centre of the Hospital of Coimbra University (CHUC) database during the period 2009–2013. More details about the rationale and the design of the database can be found in Khalighi et al. [38]. The database is publicly accessible online [39].

### Neural network architecture

**Fig 1:**
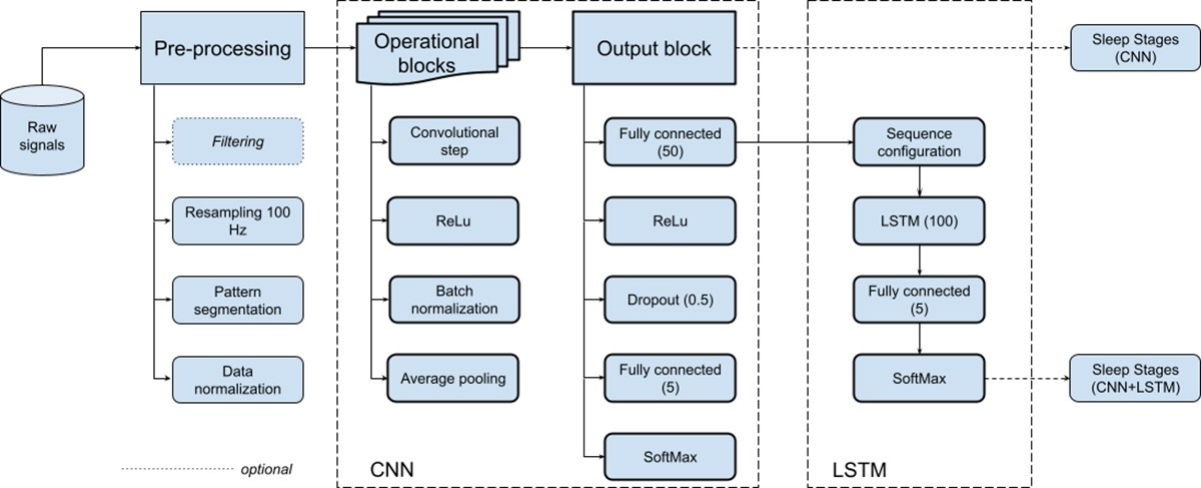
Preprocessing steps and general CNN-LSTM neural network architecture.

Here we describe the general deep learning architecture proposed for the implementation of an automatic sleep staging model. As illustrated in Fig 1, the general architecture is composed of three main processing modules: (i) pre-processing block, (ii) Convolutional Neural Network (CNN), and (iii) a Long-Short Term Memory (LSTM).

#### Pre-processing

The preprocessing block is in charge of processing the PSG signals for input homogenization and for (optionally) artifact cancellation. Input signal homogenization is necessary to confer the model the capacity to handle inter-database differences due to the use of different montages and digitalization procedures. Specifically the model receives as input two EEG, the chin EMG, and one EOG channel derivations, which are resampled at 100 Hz, representing a compromise between the limiting the size of the input dimensionality, and the preservation of the necessary signal properties for carrying out the sleep scoring task. Resampling at 100 Hz allows a working frequency up to 50 Hz which captures most of the meaningful EEG, EMG and EOG frequencies. Signals are then segmented using a 30s window following the standard epoch-based scoring procedures [1], resulting on input patterns of size 4x3000 that are fed into the following CNN processing block. Each of these input patterns is subsequently normalized in amplitude using a Gaussian standardization procedure [40].

Input signal filtering is left as an optional pre-processing step. The main purpose of this module is the removal of noise and signal artifacts, which are patient and database specific, and thus can interfere with the generalization capabilities of the resulting model. Application of the optional filtering step takes place over the original raw signals, i.e. at the original signal frequencies before resampling them at 100 Hz. Experimentation is carried out in this work to study the effects of applying the following pre-processing step on the different tested datasets. The filtering step is composed of the following filters:

- Notch filtering: It is meant to remove the interference caused by the power grid. Notice that the AC frequency differs per country (e.g. 50 Hz in Europe, and 60 Hz in North America) and therefore, depending on the source dataset, mains interference will affect signals at different frequency ranges. Design and implementation of the used digital filter has been described in previous works [41,42].
- High-pass filter: It is applied to the chin EMG only, and the purpose is to get rid of the DC and low frequency components unrelated to the baseline muscle activity. A first order implementation has been described elsewhere [42]. In this work a cut-off value at 15 Hz has been used for the filter.
- ECG filtering: Applied only in the case that an additional ECG derivation is included in the corresponding montage (see S1 Table) the filter is used for getting rid of possible spurious twitches caused by the ECG, affecting the input signals. An adaptive filtering algorithm has been used which has been described in detail in a previous work [41].

#### CNN block

The CNN block design is an updated version of previous CNN models developed by the authors [19,24]. As stated before, this block receives input patterns of size 4x3000, representing a 30s epoch window of PSG signals (2xEEG, 1xEMG, and 1xEOG). The block can produce a valid sleep staging output for each input pattern (CNN-only), or act as intermediate processing layer to feed a subsequent LSTM block (CNN-LSTM configuration). Experimentation will be carried out in this work to compare the two possible neural network configurations.

The CNN design is composed of the concatenation of *N* operational blocks. Each operational block *B(k)*, *k* = *1*…*N*, is at the same time composed of four layers, namely (*i*) a 1D convolutional step (kernel size 1x100, preserving the input size with zero padding at edges, stride = 1), followed by (*ii*) ReLu activation [43], (*iii*) batch normalization [44], and (*iv*) an average pool layer (pool dimension 1x2, stride = 2). While the kernel size (1x100) at the convolutional step is maintained through all the *N* operational blocks, the number of filters in *B(k)*, is doubled as with respect to *B(k-1)*. Based on previous experiments [19] the initial number of filters in *B(1)* was set for this work at 8, while the number of operational blocks was fixed at *N* = 3.

Output of the last operational block is fed into a subsequent CNN output block. The first processing layer in the output block is a full-connected step which takes the output from the last operational block and reduces the feature space to an output size of 50. This will be used as the input for the subsequent LSTM processing block when the network is working under the CNN-LSTM configuration. When the network is configured as CNN-only, then four additional processing steps follow. Specifically the 50-length feature vector is filtered through an additional ReLu activation, and then a dropout step with probability 0.5 is applied to improve regularization. Finally a final dense full-connected layer with *softmax* activation is used at the output with size 5, each representing a possible sleep stage assignment (W, N1, N2, N3, or R). The output of the *softmax* is interpreted as the corresponding posterior class probability, with the highest probability determining the final classification decision.

#### LSTM block

When the network follows the CNN-LSTM configuration, the 50-length feature vector is fed into a subsequent LSTM processing block. The inclusion of an additional LSTM layer in the design is meant to provide the resulting network with the capacity of modelling the effect of epoch sequence on the final scoring. Indeed, the medical expert decision on the classification of the current PSG epoch is partially influenced by the sleep state of the preceding and subsequent epochs [1].

The LSTM block is composed of a first sequence configuration layer, a unidirectional LSTM layer [45], and finally, a fully-connected layer followed by *softmax* activation for producing the final output. The sequence configuration step composes the corresponding epoch feature sequence relative to the epoch *k* under evaluation. Specifically given a PSG recording containing *M* epoch intervals, for a given epoch *k*, *k* = 1…*M*, the sequence *S(k)* is composed as $\left[F\left(k-\lceil \frac{L-1}{2}\rceil \right),F\left(k+1-\lceil \frac{L-1}{2}\rceil \right),\ldots ,F(k-1),F(k),F(k+1),\ldots ,F\left(k-1+\lceil \frac{L-1}{2}\rceil \right),F(k+\left\lfloor \frac{L-1}{2}\right\rfloor )\right]$, where ⌈⌉ and ⌊⌋ respectively represent the *ceil* and the *floor* operations, *L* is the length of the sequence, and *F* stands for the corresponding input feature vector, in this case out of the preceding CNN node. For example, if *L* = 3, then the sequence would result as [*F(k-1)*, *F(k)*, *F(K+1)*], and if *L* = 4, then [*F(k-2)*, *F(k-1)*, *F(k)*, *F(k+1)*], and so on. The number of hidden neurons for the LSTM layer was set to 100 in this study.

### Ensemble of local models

The intuitive approach to achieve better generalization of a machine learning model is to increment the amount and heterogeneity of the input training data. In the scenario where data from different sources (in our case, different databases) are involved, the former would translate into using data from the all the available datasets. Thereby the amount of training data increases, as well as their heterogeneity, hence boosting the chances of ending up with a better generalist model minimizing the dataset overfitting risk. This approach, however, has its own drawbacks. First, from a computational perspective, higher memory and computational resources are needed, the resulting model becomes inflexible to data evolving dynamically in time, and a combinatory explosion occurs when finding the best input dataset partition combination [24]. In addition, from a regulatory perspective, collecting data from different centers can be a problem due to potential privacy-protection restrictions on exchanging of patient data.

At this respect a proposal was depicted on a previous work [24] based on the use of an ensemble of local models. Under this approach an independent “local” model is developed for each dataset using exclusively its data. For this purpose each dataset is split whereby part of the data are used for training and parameterization of the machine learning model, and the remaining are set aside to be used as independent local testing set. The resulting individual local models can be then combined using an ensemble. Specifically, in this work we are assuming that the ensemble output takes place using the majority vote [46,47].

The proposed approach shows advantages in the scalability of the design, making it flexible to dynamic evolution of the input datasets, i.e. the ensemble can be easily expanded by adding new local models when new training data, or new datasets are available. This, in addition, allows each individual model to be developed locally, meaning each center can develop its own model based on its data without the need of sharing and/or collecting data from other centers. This minimizes potential issues due to patient privacy protection regulations. Eventually only the resulting local model would need to be shared for its integration in the final ensemble.

In this study we want to check the working hypothesis that by combining “local expert models” by means of an ensemble we can also increase the overall generalization capabilities of the resulting model when predicting external datasets.

### Experimental design

An experimental design was scheduled aimed at testing the prediction and generalization capabilities of the deep learning architecture for automatic sleep staging described in the preceding sections. In order to characterize the effects on generalization performance due to varying characteristics of the target database, validation was carried out on a multiple-database setup. Experiments were designed to assess and compare both independent local and external database prediction scenarios separately.

No *a posteriori* exclusion criteria were applied on any of the benchmark datasets used for this study. Thus, all the recordings integrating the datasets as described above were included in the validation. The underlying motivation is to assess the reliability of the resulting models on the most realistic situation, including the most general and heterogeneous patient phenotype possible.

Remarkably, signal montages, recording methods, and manual scoring references can differ across the different source databases. That represents an extra challenge on testing the generalization capabilities of the sleep scoring algorithm. As stated before, our deep learning model assumes as input two channels of EEG, one submental EMG, and one EOG derivation. When more than two EEG derivations were available in the corresponding montage, the general rationale was to select the traditional central derivations (C4/M1 and C3/M2) as input. If central derivations were not available, then frontal electrodes were used as backup. In some cases, no choice was possible according to this rationale, therefore the only available derivations must to be used (e.g. for Telemetry, Pz-Oz and Fpz-Cz). In the case of the EOG, horizontal derivations were preferred as they are less sensitive to EEG and movement artifacts. S1 Table describes the specific selected derivations according to the available set of channels as well as the main characteristics for each dataset.

The current AASM scoring standard [1] was set as reference for labelling the output classes for validation. Hence, when the reference dataset was originally scored using the R&K method (see S1 Table), NREM stages 3 and 4 were merged into one unique N3.

For each dataset *k*, *k = 1*…*K*, the following experiments are carried out:

Experiment 1:

- Each dataset *k*, is split following an independent training *TR(k)* and testing *TS(k)* partition. Let us denote the whole original dataset by *W(k) = TR(k)∪ TS(k)*. A model *M(k)* is derived by learning from data in *TR(k)*. Notice that a subset of *TR(k)*–namely the validation subset *VAL(k)*- is used to implement the early stopping criterion during the network’s learning process. The “local” generalization performance of the resulting model *M(k)* is evaluated by assessing the predictability of data contained in *TS(k)*. This is the performance that is usually reported in the literature when data from only one database is used for experimentation.

Experiment 2:

- Each resulting model *M(k)*, is used to predict the reference scorings on each of the complete datasets *W(j)*, *j = 1*…*K*. Effectively, ∀*j*/*j*<>*k*, *M(k)* is predicting unseen data from an external database. Hence, by comparing the results of *Experiment 1* and *Experiment 2*, the effects of varying the database target can be assessed. In effect, for each *M(k)*, the expected local generalization in *TS(k)* can be compared with the effective inter-database generalization performance among all W(*j*), *j<>k*. Notice that when *j = k* the results would be biased since *TR(k)* = > *M(k)* and *TR*(*k*)⊆*W*(*k*).

Experiment 3:

- Each dataset *W(k)* is predicted by an ensemble *ENS(k)* of individual local models *M(j)*, *j = 1*…*K*, *j<>k*. For instance, *ENS(2)* = *ENS*[*M(1)*, *M(3)*,*…*, *M(K)*]. As in Experiment 2, exclusion of *M(k)* from *ENS(k)* aims to keep *W(k)* completely independent and external to *ENS(k)*. By comparing the results of *Experiment 3* with those of *Experiment 1* and *Experiment 2*, it is possible to assess the effects on the resulting inter-database generalization of the proposed ensemble approach.

Each of the previously described experiments is repeated using different variations of the general network architecture described in the preceding sections. The purpose is to analyze the impact of each configuration variation on the resulting generalization capabilities of the resulting models. Specifically, the following variants are tested:

- Using the CCN-only configuration, first the default segments of 30s (1 epoch, input size 4x3000) configure the input to the network’s *CNN block*. The input segments are afterwards expanded to form sequences of consecutive epochs with the aim of implementing the effect of epoch sequence learning. Different sequence lengths *L* = {3,5,7} are investigated at this respect. Gaussian normalization takes place in this case over the whole 4x(3000L) resulting input patterns. This approach to implement epoch sequence learning using a CCN-only configuration will be later on compared with the results achieved using the full CNN-LSTM design.
- Using CNN-LSTM configuration, the sequence length parameter is similarly tested on different values *L* = {3,5,7}, using as input reference the 50-length feature vector of the preceding CNN output block. As stated before, the resulting models will be compared against the respective sequence learning implementations using the CNN-only configuration.
- Finally, in order to test the effects of the optional signal preprocessing filtering step, each of the previous described experiments is repeated again, respectively, with and without applying the filtering pipeline.

Thus, for each of the datasets included in our experimentation, a total of 14 different individual local models are developed, based on the data contained on each respective dataset. For identification, the following nomenclature is used: *CNN_1*, *CNN_3*, *CNN_5*, *CNN_7*, *CNN_F_1*, *CNN_F_3*, *CNN_F_5*, *CNN_F_7*, *CNN_LSTM_3*, *CNN_LSTM_5*, *CNN_LSTM_7*, *CNN_LSTM_F_3*, *CNN_LSTM_F_5*, *CNN_LSTM_F_7*, where the subscript *F* denotes the use of the pre-processing filtering step, and the suffix number indicates the corresponding number of sequence epochs used (value of the *L* parameter).

For homogenization purposes, the same training configuration is applied in the development of above mentioned learning models for each dataset. In this respect the stochastic gradient descent approach is used to guide the weight’s update, with the cross-entropy loss as the target cost function [40]. Each dataset is partitioned using 80% of data for training (TR), using the remaining 20% as independent local testing set (TS). A validation subset (VAL) is arranged by successively splitting 20% of the available training data apart. The validation set is used as reference to implement the early stopping mechanism to avoid overfitting to training data. The stopping criterion takes as reference the validation loss, which is evaluated 5 times per training epoch. A patience of 10 is established thereby stopping training when the validation loss has not been further improved after the whole training dataset is presented two times. The number of patterns within each training epoch (internal training batch) is set to 100 patterns, imposed by the available hardware resources relative to the size of the tested networks. The maximum number of training epochs is set to 30, and the initial learning rate to 0.001. The learning rate is decreased by a factor of 10 every 10 training epochs (thus 10^−4^, 10^−5^, up to a minimum of 10^−6^). The same random initialization seed is used on each experiment to exclude variability due to initialization conditions, hence enabling deterministic training processes. This is important to assess the influence of the different tested architecture variants, as described before, and to make fair comparisons among the different resulting models and datasets.

Performance evaluation of each experiment is carried out by taking the Cohen’s kappa index (κ) as reference score. Cohen’s kappa is preferred over other widespread validation metrics (e.g. accuracy, sensitivity/specificity, or *F*_*1*_-score) because it corrects for agreement due to chance, showing robustness in the presence of various class distributions [48]. This is an important property to allow performance comparison among differently distributed datasets, or when some classes are underrepresented in proportion to the rest (e.g. N1 vs N2 or W), as it is the case (see S1 Table for details on the different class distributions among the benchmark datasets). Remarkably, Cohen´s kappa is the standard metric being reported among studies analyzing human inter-rater agreement in the context of sleep scoring [49–54].

## Results

The following tables contain the results of the experiments described in the previous sections.

Table 1 shows the results of Experiment 1, where each of the learning models is trained and evaluated using data from its respective local testing dataset.

**Table 1:** Performance results of each individual model on the local validation scenario.

Local dataset	Model configuration	Training iterations	TR	VAL	TS
HMC	CNN_1	15	0.79	0.73	0.74
	CNN_3	7	0.83	0.72	0.71
	CNN_5	7	0.87	0.71	0.7
	CNN_7	5	0.83	0.69	0.69
	CNN_LSTM_3	7	0.81	0.78	0.78
	CNN_LSTM_5	17	0.84	0.79	0.79
	CNN_LSTM_7	27	0.83	0.77	0.77
	CNN_F_1	14	0.78	0.73	0.74
	CNN_F_3	8	0.84	0.71	0.71
	CNN_F_5	6	0.84	0.7	0.7
	CNN_F_7	5	0.85	0.69	0.69
	CNN_LSTM_F_3	7	0.79	0.77	0.77
	CNN_LSTM_F_5	8	0.77	0.75	0.75
	CNN_LSTM_F_7	10	0.76	0.74	0.74
Dublin	CNN_1	10	0.76	0.68	0.68
	CNN_3	7	0.85	0.66	0.66
	CNN_5	6	0.89	0.62	0.64
	CNN_7	7	0.89	0.65	0.67
	CNN_LSTM_3	8	0.82	0.76	0.77
	CNN_LSTM_5	9	0.84	0.78	0.79
	CNN_LSTM_7	9	0.84	0.77	0.77
	CNN_F_1	14	0.77	0.65	0.65
	CNN_F_3	8	0.83	0.65	0.64
	CNN_F_5	8	0.88	0.6	0.61
	CNN_F_7	13	0.9	0.67	0.66
	CNN_LSTM_F_3	8	0.81	0.76	0.77
	CNN_LSTM_F_5	8	0.82	0.78	0.79
	CNN_LSTM_F_7	9	0.84	0.77	0.78
SHHS	CNN_1	9	0.8	0.76	0.75
	CNN_3	7	0.89	0.79	0.79
	CNN_5	7	0.95	0.78	0.79
	CNN_7	6	0.92	0.76	0.76
	CNN_LSTM_3	17	0.87	0.83	0.84
	CNN_LSTM_5	18	0.86	0.83	0.82
	CNN_LSTM_7	7	0.79	0.78	0.77
	CNN_F_1	9	0.8	0.77	0.76
	CNN_F_3	8	0.89	0.79	0.79
	CNN_F_5	5	0.9	0.78	0.78
	CNN_F_7	6	0.94	0.78	0.77
	CNN_LSTM_F_3	10	0.85	0.82	0.83
	CNN_LSTM_F_5	18	0.86	0.83	0.82
	CNN_LSTM_F_7	5	0.8	0.79	0.79
Telemetry	CNN_1	14	0.81	0.76	0.76
	CNN_3	10	0.88	0.73	0.75
	CNN_5	8	0.9	0.73	0.72
	CNN_7	6	0.88	0.72	0.7
	CNN_LSTM_3	10	0.85	0.8	0.81
	CNN_LSTM_5	9	0.85	0.79	0.8
	CNN_LSTM_7	8	0.84	0.8	0.8
	CNN_F_1	14	0.82	0.77	0.77
	CNN_F_3	8	0.87	0.71	0.71
	CNN_F_5	9	0.88	0.73	0.73
	CNN_F_7	9	0.91	0.73	0.71
	CNN_LSTM_F_3	10	0.84	0.81	0.81
	CNN_LSTM_F_5	15	0.89	0.82	0.83
	CNN_LSTM_F_7	16	0.89	0.82	0.82
DREAMS	CNN_1	9	0.81	0.75	0.76
	CNN_3	8	0.92	0.76	0.76
	CNN_5	7	0.91	0.75	0.75
	CNN_7	5	0.85	0.73	0.73
	CNN_LSTM_3	17	0.88	0.84	0.83
	CNN_LSTM_5	20	0.9	0.83	0.83
	CNN_LSTM_7	5	0.81	0.78	0.78
	CNN_F_1	9	0.82	0.76	0.77
	CNN_F_3	7	0.91	0.77	0.78
	CNN_F_5	7	0.92	0.74	0.75
	CNN_F_7	6	0.88	0.73	0.72
	CNN_LSTM_F_3	20	0.89	0.84	0.83
	CNN_LSTM_F_5	28	0.9	0.84	0.84
	CNN_LSTM_F_7	10	0.85	0.81	0.8
ISRUC	CNN_1	17	0.81	0.77	0.76
	CNN_3	6	0.83	0.74	0.75
	CNN_5	7	0.9	0.73	0.73
	CNN_7	6	0.86	0.72	0.73
	CNN_LSTM_3	10	0.81	0.8	0.8
	CNN_LSTM_5	10	0.8	0.78	0.78
	CNN_LSTM_7	6	0.75	0.75	0.75
	CNN_F_1	9	0.79	0.76	0.75
	CNN_F_3	7	0.84	0.75	0.75
	CNN_F_5	7	0.9	0.73	0.73
	CNN_F_7	6	0.86	0.71	0.72
	CNN_LSTM_F_3	10	0.81	0.79	0.79
	CNN_LSTM_F_5	9	0.78	0.77	0.76
	CNN_LSTM_F_7	9	0.75	0.74	0.74

Results report agreement in terms of kappa index with respect to the corresponding human clinical scorings for each dataset. Agreement is reported separately for each corresponding training (TR), validation (VAL) and testing (TS) dataset partitions. The number of effective training iterations is indicated in the third column. Rows within each dataset correspond to the different tested neural network configurations as described in the experimental design.

Subsequent Table 2 shows the results of the second experiment in which the resulting individual local models have been used to predict the reference scorings on each of the complete datasets. Results in Table 2 therefore involve performance evaluations of the models using an external validation setting, with the only exception of the main diagonal. The main diagonal in Table 2 represents the situation in which *M(k)* is used to predict *W(k)*, resulting in a biased prediction since *TR(k)* = > *M(k)* and *TR*(*k*)⊆*W*(*k*). Regardless, these results have been kept in Table 2 for reference.

**Table 2:** Performance results of the individual local models on the external validation scenario.

		Individual local models					
Predicted dataset	Model configuration	M(HMC)	M(Dublin)	M(SHHS)	M(Telemetry)	M(DREAMS)	M(ISRUC)
HMC	CNN_1	0.77	0.51	0.56	0.53	0.52	0.6
	CNN_3	0.79	0.46	0.6	0.42	0.47	0.56
	CNN_5	0.81	0.37	0.57	0.39	0.44	0.54
	CNN_7	0.78	0.4	0.5	0.34	0.43	0.55
	CNN_LSTM_3	0.8	0.54	0.58	0.51	0.5	0.61
	CNN_LSTM_5	0.82	0.53	0.6	0.5	0.49	0.62
	CNN_LSTM_7	0.81	0.52	0.59	0.52	0.51	0.61
	CNN_F_1	0.76	0.39	0.58	0.49	0.56	0.62
	CNN_F_3	0.79	0.37	0.6	0.48	0.48	0.63
	CNN_F_5	0.79	0.35	0.57	0.42	0.46	0.61
	CNN_F_7	0.79	0.35	0.54	0.37	0.5	0.58
	CNN_LSTM_F_3	0.78	0.38	0.62	0.45	0.55	0.64
	CNN_LSTM_F_5	0.76	0.37	0.63	0.48	0.55	0.65
	CNN_LSTM_F_7	0.75	0.34	0.62	0.47	0.55	0.62
Dublin	CNN_1	0.53	0.73	0.44	0.41	0.53	0.51
	CNN_3	0.57	0.78	0.5	0.34	0.49	0.57
	CNN_5	0.52	0.79	0.51	0.32	0.51	0.59
	CNN_7	0.53	0.81	0.39	0.31	0.49	0.57
	CNN_LSTM_3	0.54	0.8	0.48	0.38	0.58	0.55
	CNN_LSTM_5	0.54	0.82	0.5	0.39	0.58	0.55
	CNN_LSTM_7	0.5	0.81	0.5	0.42	0.57	0.55
	CNN_F_1	0.2	0.73	0.13	0.03	0.01	0.07
	CNN_F_3	0.15	0.77	0.1	0.01	0.02	0.05
	CNN_F_5	0.04	0.78	0.02	0.01	0.03	0.04
	CNN_F_7	0.04	0.81	0.13	0.01	0.02	0.14
	CNN_LSTM_F_3	0.24	0.8	0.07	0.02	0.01	0.05
	CNN_LSTM_F_5	0.22	0.81	0.07	0.01	0.01	0.05
	CNN_LSTM_F_7	0.17	0.81	0.06	0.01	0.01	0.04
SHHS	CNN_1	0.57	0.5	0.78	0.42	0.59	0.64
	CNN_3	0.59	0.52	0.86	0.3	0.6	0.63
	CNN_5	0.55	0.42	0.89	0.27	0.6	0.63
	CNN_7	0.61	0.4	0.86	0.26	0.6	0.65
	CNN_LSTM_3	0.54	0.57	0.86	0.42	0.54	0.62
	CNN_LSTM_5	0.5	0.56	0.85	0.46	0.52	0.67
	CNN_LSTM_7	0.47	0.53	0.78	0.46	0.56	0.66
	CNN_F_1	0.68	0.35	0.78	0.43	0.6	0.65
	CNN_F_3	0.53	0.29	0.85	0.38	0.59	0.65
	CNN_F_5	0.52	0.32	0.86	0.39	0.58	0.68
	CNN_F_7	0.52	0.28	0.88	0.31	0.63	0.67
	CNN_LSTM_F_3	0.68	0.29	0.84	0.4	0.57	0.63
	CNN_LSTM_F_5	0.66	0.31	0.85	0.39	0.53	0.65
	CNN_LSTM_F_7	0.67	0.22	0.79	0.41	0.57	0.62
Telemetry	CNN_1	0.67	0.53	0.51	0.79	0.48	0.63
	CNN_3	0.6	0.42	0.55	0.81	0.43	0.53
	CNN_5	0.6	0.43	0.54	0.83	0.48	0.57
	CNN_7	0.5	0.45	0.38	0.82	0.45	0.52
	CNN_LSTM_3	0.68	0.59	0.49	0.83	0.45	0.62
	CNN_LSTM_5	0.69	0.59	0.5	0.83	0.43	0.64
	CNN_LSTM_7	0.67	0.61	0.5	0.82	0.48	0.65
	CNN_F_1	0.7	0.39	0.61	0.8	0.44	0.61
	CNN_F_3	0.67	0.46	0.57	0.83	0.46	0.62
	CNN_F_5	0.63	0.43	0.41	0.83	0.42	0.55
	CNN_F_7	0.64	0.44	0.33	0.84	0.46	0.54
	CNN_LSTM_F_3	0.72	0.44	0.6	0.83	0.43	0.6
	CNN_LSTM_F_5	0.71	0.48	0.62	0.87	0.44	0.63
	CNN_LSTM_F_7	0.68	0.44	0.6	0.86	0.44	0.61
DREAMS	CNN_1	0.5	0.56	0.58	0.34	0.79	0.71
	CNN_3	0.46	0.52	0.59	0.34	0.86	0.71
	CNN_5	0.42	0.33	0.36	0.31	0.85	0.68
	CNN_7	0.61	0.51	0.58	0.27	0.81	0.67
	CNN_LSTM_3	0.5	0.56	0.54	0.43	0.87	0.73
	CNN_LSTM_5	0.41	0.56	0.54	0.47	0.87	0.75
	CNN_LSTM_7	0.45	0.5	0.51	0.42	0.8	0.74
	CNN_F_1	0.52	0.39	0.66	0.42	0.8	0.7
	CNN_F_3	0.59	0.25	0.56	0.46	0.86	0.67
	CNN_F_5	0.55	0.36	0.55	0.4	0.86	0.65
	CNN_F_7	0.52	0.32	0.58	0.35	0.82	0.71
	CNN_LSTM_F_3	0.53	0.32	0.6	0.43	0.87	0.71
	CNN_LSTM_F_5	0.55	0.31	0.63	0.46	0.88	0.72
	CNN_LSTM_F_7	0.51	0.16	0.6	0.43	0.83	0.71
ISRUC	CNN_1	0.56	0.57	0.6	0.29	0.63	0.79
	CNN_3	0.57	0.54	0.64	0.29	0.56	0.8
	CNN_5	0.51	0.46	0.63	0.26	0.57	0.84
	CNN_7	0.57	0.48	0.54	0.24	0.52	0.81
	CNN_LSTM_3	0.54	0.55	0.65	0.36	0.61	0.81
	CNN_LSTM_5	0.51	0.55	0.66	0.42	0.58	0.79
	CNN_LSTM_7	0.43	0.53	0.6	0.38	0.6	0.75
	CNN_F_1	0.68	0.42	0.63	0.42	0.65	0.77
	CNN_F_3	0.59	0.35	0.65	0.41	0.61	0.81
	CNN_F_5	0.57	0.41	0.66	0.37	0.57	0.84
	CNN_F_7	0.55	0.38	0.62	0.35	0.56	0.81
	CNN_LSTM_F_3	0.68	0.41	0.68	0.4	0.63	0.8
	CNN_LSTM_F_5	0.67	0.43	0.69	0.44	0.61	0.78
	CNN_LSTM_F_7	0.66	0.29	0.66	0.43	0.61	0.75

Results report agreement in terms of kappa index with respect to the corresponding human clinical scorings for each dataset. The notation M(X) is used to indicate that the model was trained based on data on the dataset X. Rows within each dataset correspond to the different tested neural network configurations as described in the experimental design. The main diagonal (in greyed background) shows the results when the model is predicting its own complete local dataset (biased prediction).

Results regarding the third experiment (ensemble predictions) are shown in Table 3. These are compared with the reference predictions of the individual local models, both in the local and external validation scenarios. The third column in Table 3 shows the reference local predictions achieved by the models in their respective testing sets (last column of Table 1). Subsequently, the fourth column shows the corresponding ranges of the inter-database external predictions as derived from data in Table 2. These ranges exclude data from the main diagonal of Table 2, i.e. for dataset *k*, performance of *M(k)* is excluded, hence regarding performance when the individual models are presented with the target dataset on an external prediction scenario exclusively. The resulting average performance is shown in the fifth column. Finally, the last column of Table 3 shows the corresponding performance when the ensemble model is used for predicting the corresponding dataset. Similarly, *ENS(k)* excludes *M(k)* from the ensemble, e.g. for HMC, the derived predictions result from *ENS*[*M(Dublin)*, *M(SHHS)*, *M(Telemetry)*, *M(DREAMS)*, *M(ISRUC)*], and so for.

**Table 3:** Performance comparison between individual models and the ensemble approach in the local and external validation scenarios.

Predicted dataset	Model configuration	Individual local models			Ensemble
		Local performance	External performance		External performance
			Range	Average	
HMC	CNN_1	0.74	0.51–0.60	0.54	0.61
	CNN_3	0.71	0.42–0.60	0.5	0.58
	CNN_5	0.7	0.37–0.57	0.46	0.55
	CNN_7	0.69	0.34–0.55	0.44	0.53
	CNN_LSTM_3	0.78	0.50–0.61	0.55	0.62
	CNN_LSTM_5	0.79	0.49–0.62	0.55	0.63
	CNN_LSTM_7	0.77	0.51–0.61	0.55	0.62
	CNN_F_1	0.74	0.39–0.62	0.53	0.61
	CNN_F_3	0.71	0.37–0.63	0.51	0.6
	CNN_F_5	0.7	0.35–0.61	0.48	0.58
	CNN_F_7	0.69	0.35–0.58	0.47	0.56
	CNN_LSTM_F_3	0.77	0.38–0.64	0.53	0.62
	CNN_LSTM_F_5	0.75	0.37–0.65	0.54	0.64
	CNN_LSTM_F_7	0.74	0.34–0.62	0.52	0.63
Dublin	CNN_1	0.68	0.41–0.53	0.49	0.6
	CNN_3	0.66	0.34–0.57	0.49	0.62
	CNN_5	0.64	0.32–0.59	0.49	0.6
	CNN_7	0.67	0.31–0.57	0.46	0.59
	CNN_LSTM_3	0.77	0.38–0.58	0.51	0.63
	CNN_LSTM_5	0.79	0.39–0.58	0.51	0.63
	CNN_LSTM_7	0.77	0.42–0.57	0.51	0.62
	CNN_F_1	0.65	0.01–0.20	0.09	0.08
	CNN_F_3	0.64	0.01–0.15	0.07	0.04
	CNN_F_5	0.61	0.01–0.04	0.03	0.01
	CNN_F_7	0.66	0.01–0.14	0.07	0.03
	CNN_LSTM_F_3	0.77	0.01–0.24	0.08	0.06
	CNN_LSTM_F_5	0.79	0.01–0.22	0.07	0.05
	CNN_LSTM_F_7	0.78	0.01–0.17	0.06	0.04
SHHS	CNN_1	0.75	0.42–0.64	0.54	0.62
	CNN_3	0.79	0.30–0.63	0.53	0.65
	CNN_5	0.79	0.27–0.63	0.49	0.61
	CNN_7	0.76	0.26–0.65	0.5	0.65
	CNN_LSTM_3	0.84	0.42–0.62	0.54	0.62
	CNN_LSTM_5	0.82	0.46–0.67	0.54	0.61
	CNN_LSTM_7	0.77	0.46–0.66	0.54	0.61
	CNN_F_1	0.76	0.35–0.68	0.54	0.66
	CNN_F_3	0.79	0.29–0.65	0.49	0.62
	CNN_F_5	0.78	0.32–0.68	0.5	0.62
	CNN_F_7	0.77	0.28–0.67	0.48	0.62
	CNN_LSTM_F_3	0.83	0.29–0.68	0.52	0.62
	CNN_LSTM_F_5	0.82	0.31–0.66	0.51	0.62
	CNN_LSTM_F_7	0.79	0.22–0.67	0.5	0.62
Telemetry	CNN_1	0.76	0.48–0.67	0.56	0.67
	CNN_3	0.75	0.42–0.60	0.51	0.61
	CNN_5	0.72	0.43–0.60	0.53	0.62
	CNN_7	0.7	0.38–0.52	0.46	0.58
	CNN_LSTM_3	0.81	0.45–0.68	0.57	0.67
	CNN_LSTM_5	0.8	0.43–0.69	0.57	0.69
	CNN_LSTM_7	0.8	0.48–0.67	0.58	0.68
	CNN_F_1	0.77	0.39–0.70	0.55	0.66
	CNN_F_3	0.71	0.46–0.67	0.56	0.66
	CNN_F_5	0.73	0.41–0.63	0.49	0.62
	CNN_F_7	0.71	0.33–0.64	0.48	0.63
	CNN_LSTM_F_3	0.81	0.43–0.72	0.56	0.69
	CNN_LSTM_F_5	0.83	0.44–0.71	0.58	0.7
	CNN_LSTM_F_7	0.82	0.44–0.68	0.56	0.68
DREAMS	CNN_1	0.76	0.34–0.71	0.54	0.61
	CNN_3	0.76	0.34–0.71	0.52	0.61
	CNN_5	0.75	0.31–0.68	0.42	0.56
	CNN_7	0.73	0.27–0.67	0.53	0.63
	CNN_LSTM_3	0.83	0.43–0.73	0.55	0.61
	CNN_LSTM_5	0.83	0.41–0.75	0.55	0.59
	CNN_LSTM_7	0.78	0.42–0.74	0.52	0.58
	CNN_F_1	0.77	0.39–0.70	0.54	0.62
	CNN_F_3	0.78	0.25–0.67	0.51	0.66
	CNN_F_5	0.75	0.36–0.65	0.5	0.64
	CNN_F_7	0.72	0.32–0.71	0.5	0.63
	CNN_LSTM_F_3	0.83	0.32–0.71	0.52	0.61
	CNN_LSTM_F_5	0.84	0.31–0.72	0.54	0.64
	CNN_LSTM_F_7	0.8	0.16–0.71	0.48	0.59
ISRUC	CNN_1	0.76	0.29–0.63	0.53	0.59
	CNN_3	0.75	0.29–0.64	0.52	0.61
	CNN_5	0.73	0.26–0.63	0.48	0.58
	CNN_7	0.73	0.24–0.57	0.47	0.56
	CNN_LSTM_3	0.8	0.36–0.65	0.54	0.6
	CNN_LSTM_5	0.78	0.42–0.66	0.54	0.61
	CNN_LSTM_7	0.75	0.38–0.60	0.51	0.57
	CNN_F_1	0.75	0.42–0.68	0.56	0.64
	CNN_F_3	0.75	0.35–0.65	0.52	0.63
	CNN_F_5	0.73	0.37–0.66	0.51	0.6
	CNN_F_7	0.72	0.35–0.62	0.49	0.58
	CNN_LSTM_F_3	0.79	0.40–0.68	0.56	0.65
	CNN_LSTM_F_5	0.76	0.43–0.69	0.57	0.66
	CNN_LSTM_F_7	0.74	0.29–0.66	0.53	0.64

Results report agreement in terms of kappa index with respect to the corresponding human clinical scorings for each dataset. Rows within each dataset correspond to the different tested neural network configurations as described in the experimental design.

Finally, Table 4 shows the global results by aggregating performance of the respective models across all the tested datasets. Specifically, each row in the second, third, and fourth columns of Table 4 is calculated by averaging the corresponding rows of columns three, five, and six, in Table 3, i.e. across all the six datasets. Columns five, six, and seven in Table 4 respectively represent the averaged inter-database performance differences between *(i)* the individual models in their respective local testing datasets and their averaged external dataset predictions, *(ii)* the individual models in their respective local testing datasets and the prediction of the ensemble model, and *(iii)* the averaged external dataset predictions of the individual models and the corresponding ensemble model prediction.

**Table 4:** Global performance comparison by aggregating results across all datasets.

Model configuration	Individual models—local dataset (I)	Individual models—external datasets (II)	Ensemble—external dataset (III)	I vs II differences	I vs III differences	II vs III differences
CNN_1	0.7417	0.5333	0.6167	-0.2083	-0.125	0.0833
CNN_3	0.7367	0.5117	0.6133	-0.225	-0.1233	0.1017
CNN_5	0.7217	0.4783	0.5833	-0.2433	-0.1383	**0.105**
CNN_7	0.7133	0.485	0.59	-0.2283	-0.1233	**0.105**
CNN_LSTM_3	0.7967	**0.5433**	0.625	-0.2533	-0.1717	0.0817
CNN_LSTM_5	**0.8017**	**0.5433**	**0.6267**	-0.2583	-0.175	0.0833
CNN_LSTM_7	0.7733	0.535	0.6133	-0.2383	-0.16	0.0783
CNN_F_1	0.74	0.4683	0.545	-0.2717	-0.195	0.0767
CNN_F_3	0.73	0.4433	0.535	-0.2867	-0.195	0.0917
CNN_F_5	0.7167	0.4183	0.5117	-0.2983	-0.205	0.0933
CNN_F_7	0.7117	0.415	0.5083	-0.2967	-0.2033	0.0933
CNN_LSTM_F_3	0.8	0.4617	0.5417	**-0.3383**	**-0.2583**	0.08
CNN_LSTM_F_5	0.7983	0.4683	0.5517	-0.33	-0.2467	0.0833
CNN_LSTM_F_7	0.7783	0.4417	0.5333	-0.3367	-0.245	0.0917

Results report average agreement in terms of kappa index with respect to the corresponding human clinical scorings for each dataset: Local testing sets using individual models (I), external datasets using individual models (II), and external datasets using an ensemble of individual models (III). Each row corresponds to the different tested neural network configurations as described in the experimental design. The highest absolute values on each column are highlighted in bold.

## Analysis of experimental data

### Best model (CNN vs CNN-LSTM)

According to Table 4, the proposed deep neural network approach achieves its best generalization performance across all the tested datasets on its *CNN_LSTM_5* architectural variant. This configuration did achieve the best overall performance both in the local as well as in the external dataset prediction scenarios. The implementation of epoch sequence learning by concatenating the LSTM processing block to the output of the preceding CNN feature output layer results on an overall improvement of the model’s performance. In general the CNN-LSTM configuration outperforms the respective CNN-only counterpart for the same sequence length at both local and external generalization scenarios. Performance improves with increasing *L*, reaching a saturation value around *L* = 5, after which generalization of the model decreases again below the validation indices obtained for *L* = 3. When using the CNN-only configuration, on the other hand, augmentation of the epoch sequence length does not translate on any network´s prediction improvement. This result for the CNN-only configuration seems to be a consequence of learning overfitting, as Table 1 shows that performance on the respective training sets nevertheless keeps improving with higher values of *L*. For the CNN-LSTM configuration, however, the trend seems to be consistent between the respective training and generalization performances.

### Signal prefiltering

Data from Table 4 seems to rather advise against the use of the optional filtering pre-processing step. A closer look to the results of Tables 1–3, however, does show an inconsistent effect across the individual tested datasets. Actually data could be regarded as inconclusive or even favorable to the use of filters, with the notable exception of the results achieved for the Dublin dataset. As evidenced by data in Tables 2 and 3, the filtering step seems to have a totally different effect on the predictability of this dataset as compared with the rest. Remarkably, however, notice that difficulties of the models in predicting Dublin’s data are only evidenced when the validation is carried out on an external prediction scenario. When using Dulin as independent local testing dataset, corresponding data in Table 1 do not show the pronounced performance decay as in the previous setting. This result evidences the database variability problem, and thus importance of expanding the validation procedures beyond the usual local testing scenario, including a sufficiently heterogeneous and independent data sample from a variety of external sources.

### Database generalization performance

Having the expanded validation scenario in mind, and attending to experimental data contained in Tables 1–4, the following general statements might be formulated:

1. The individual model’s local-dataset generalization performance overestimates the actual inter-dataset external generalization. This is a consistent result across all the tested datasets and network configurations (see Table 3). The trend is globally evidenced in Table 4 as well, as *I vs II differences* in the fifth column consistently show negative values. The downgrade in performance when evaluating external data is considerable, with associated kappa indices decreasing on the range between 0.21 up to 0.34 for the tested architectural variants.
2. The proposed ensemble method improves external inter-dataset generalization performance. This result is also consistent across all experimental simulations as evidenced in Tables 3 and 4. The improvement as with respect to the performance of the individual model’s estimations ranges between 0.08 and 0.10 on the related kappa indices (see *II vs III differences* in column 7 of Table 4).
3. Individual model’s local-dataset generalization estimation still represents an upper bound for the external inter-dataset generalization achieved by the ensemble approach. Similarly, evidence is consistent across data of Tables 3 and 4, with absolute kappa differences ranging between 0.12 and 0.26 in this case (*I vs III differences* in column six of Table 4).

### Analysis in the context of the expected human performance

Table 5 summarizes literature results reporting on the expected human inter-scorer variability for the sleep staging task. Only works reporting agreement in terms of kappa index are included. Results in Table 5 are structured depending upon if experimentation implements a local or an external validation scenario, enabling a corresponding comparison with our results. In this regard, it is interpreted that a local validation was carried out when agreement among different human scorers belonging to a same center is compared. Usually this also involves the use of their own local database as the source for comparing their scorings. External inter-rater validations, on the other hand, refer to the cases in which experts compare their scorings using an independent dataset external to their center of origin. As reference for our results the *CNN_LSTM_5* architectural variant is used, which achieved the best overall performance both in the local as well as in the external dataset prediction scenarios through our experimentation.

**Table 5:** Indices of human inter-rater agreement reported in the literature compared with the performance achieved by our proposed deep-learning approach.

Dataset	Inter-rater agreement (same center/database)	Our results (local validation)	Inter-rater agreement (different center/database)	Our results (external validation)
HMC	0.74	0.79	---	0.63
Dublin	---	0.79	---	0.63
SHHS	0.81–0.83 [52]	0.82	---	0.61
Telemetry	---	0.80	---	0.69
DREAMS	---	0.83	---	0.59
ISRUC	0.87 [38]	0.78	---	0.61
***Overall range (our testing benchmark)***	**0.74–0.87**	**0.78–0.83**	**---**	**0.59–0.69**
Other databases	0.73 [11] 0.77–0.80 [55] 0.84–0.86 [56] 0.86 [54]	---	0.46–0.89 [55] 0.72–0.75 [50] 0.62 [57] 0.76 [51] 0.68 [49] 0.63 [53] 0.58 [21] 0.75 [54] 0.66 [23]	---
***Overall range (all databases)***	**0.73–0.87**	**0.78–0.83**	**0.46–0.89**	**0.59–0.69**

Results report agreement in terms of kappa index. The CNN_LSTM_5 model is taken as reference for the results regarding our automatic approach. Overall results across databases are highlighted in bold.

Attending to data in Table 5, our results in the local database generalization scenario are in the range of the expected human agreement under similar conditions (Table 5, κ = 0.78–0.83 ours vs 0.73–0.87 reference). As per dataset, the trend holds for HMC (Table 5, κ = 0.79 vs 0.74 reference) and SHHS (Table 5, κ = 0.82 vs 0.81–0.83 reference), while for ISRUC, the automatic system performs somewhat under the expected expert levels (Table 5, κ = 0.78 vs 0.87 reference). For HMC, human reference agreement levels were estimated using a subset of five recordings that were rescored by a total of 12 clinical experts from our sleep lab. The resulting pair-wise kappa agreements between all the combinations of experts were then averaged. To minimize the possibility of a biased case representation, the five recordings were selected, out of the 154 available, using a structured approach based on their relative positioning in the human-computer kappa performance distribution (12.5, 37.5, 50, 62.5 and 87.5 percentiles), where the original clinical expert scorings were used as reference. A similar selection approach was used on a previous study of the authors for the validation of an EEG arousal detection algorithm [58]. No other studies reporting on human kappa agreement were found in the literature for the rest of the datasets used in this work.

As with respect to the external inter-database scenario, analysis of the literature shows a general decrease in human performance when compared to the respective local variability references. Specifically, two works, [54] and [55], allow comparison between local and external inter-scorer variability on the same dataset. In general results on these works follow the previously mentioned downgrading trend. In [55], however, an exception to this trend is reported in one of the two tested subgroups: 23 recordings scored using the R&K standard, and 21 recordings scored using the AASM rules. Specifically for the first subgroup of 23 recordings, inter-scorer agreement seems to actually increase among scorers coming from different centers (from κ = 0.77, when scorers belong to the same center, up to κ = 0.85–0.89 [55]). This result seems to represent an outlier, and for the second subgroup the results seem to support again the general downgrading trend reported in the literature (from κ = 0.80, when scorers belong to the same center, down to κ = 0.46–0.49 [55]).

Unfortunately, baseline levels of human agreement for the external prediction scenario cannot be determined from the current available literature for none of the databases used in this work. With that in mind, external generalization performance of our automatic scoring approach still seems to fall within the range of the expected human agreement reported for other databases (Table 5, κ = 0.59–0.69 ours vs 0.46–0.89 in general).

### Analysis in the context of other automatic approaches

In Table 6 validation results comprising other automatic approaches reported in the literature are summarized. As in the previous case, results are structured considering if the performance metrics were obtained on the basis of a local or an external validation scenario. Only studies reporting agreement in terms of kappa index were considered. As reference for our results the *CNN_LSTM_5* architectural variant is used.

**Table 6:** Indices of automatic scoring agreement reported in the literature in comparison with the results achieved by the proposed deep-learning approach.

Dataset	Local dataset prediction scenario	Our results (local dataset)	External dataset prediction scenario	Our results
				(external dataset)
HMC	0.62 [24]	0.79	0.60 [24]	0.63
Dublin	0.44 [24]	0.79	0.19 [24]	0.63
	0.84 [18]			
	0.74 [59]			
	0.66 [60]			
SHHS	0.65 [24]	0.82	0.62 [24]	0.61
	0.82 [61]		0.53–0.56 [61]	
	0.73 [62]		0.73 [62]	
	0.83 [63]		0.52–0.73 [64]	
	0.81 [64]			
Telemetry	0.58 [24]	0.8	0.53 [24]	0.69
DREAMS	0.62 [24]	0.83	0.43 [24]	0.59
ISRUC	0.68 [24]	0.78	0.63 [24]	0.61
	0.65 [64]		0.57–0.68 [64]	
***Overall range (our testing benchmark)***	**0.44–0.84**	**0.78–0.83**	**0.19–0.73**	**0.59–0.69**
Other databases	0.86 [65]	---	0.42–0.63 [55]	---
	0.76–0.80 [17]		0.68–0.70 [61]	
	0.84 [66]		0.69 [62]	
	0.80 [59]		0.72–0.77 [21]	
	0.68 [67]		0.45–0.70 [20]	
	0.81 [62]		0.61 [23]	
	0.73–0.76 [20]		0.50–0.76 [64]	
	0.82 [68]			
	0.77 [60]			
	0.66 [23]			
	0.70–0.79 [64]			
***Overall range (all databases)***	**0.44–0.86**	**0.78–0.83**	**0.19–0.77**	**0.59–0.69**

Results report agreement in terms of kappa index. The CNN_LSTM_5 model is taken as reference for the results regarding our automatic approach. Overall results across databases are highlighted in bold.

According to Table 6, when comparing local generalization performance on the datasets used in this work, our approach falls within the upper range of the corresponding state-of-the-art results (Table 6, κ = 0.78–0.83 in this work vs 0.44–0.84 overall). In particular, the architecture presented in this work clearly outperforms the previous results reported by the authors using the exact same datasets (κ = 0.44–0.68 in [24]). Other works have reported results in the case of the Dublin, SHHS, and ISRUC datasets. In the Dublin dataset our approach (κ = 0.79) outperforms results in existing literature [59,60] (κ = 0.66–0.74) but in the case of [18] (κ = 0.84). Notice [18] does not report results regarding external independent validation, and therefore overfitting to the local database should not be discarded. In the case of SHHS, our results (κ = 0.82) outperforms those reported in [62] and [64] (κ = 0.73 and 0.81, respectively) and matches those in [61]. On one another author’s previous work [63] slightly better results were reported for SHHS (κ = 0.83), however the results in [63] shared the limitation that validation was only carried out the local dataset prediction scenario. No local performance reference has been found in the literature for HMC, Telemetry, and DREAMS datasets.

When considering performance on the local dataset scenario globally, including results reported on other benchmarks, performance of our approach still holds on the upper range (Table 6, κ = 0.44–0.86 globally vs 0.78–0.83 in this work). Notice that the highest performance reported in [65] (κ = 0.86) was obtained using 50% of the data from a small dataset of 8 recordings only, also not including validation data on external datasets.

When considering data on the external dataset validation, Table 6 shows a general global decrease in the performance of the automatic methods as with respect to the corresponding indices on the local database validation scenario. Specifically, in all the works that allow comparison between local and external database generalization using the same algorithm [20,23,24,62,64] decrease in performance in noticeable when tested using external independent datasets. This trend is consistent with the results of our experimentation, as well as with data regarding human inter-rater agreement analyzed in Table 5.

Overall, the highest external database generalization performance reported in the literature has been described in [21] (κ = 0.72–0.77). Results regard the best model on a leaving-one-out consensus of experts using one independent external dataset (IS-RC, see Table 1 in [21]). Unfortunately, local generalization performance in terms of kappa was not reported for the same model in that work. Therefore, it is not possible to evaluate possible differences between local and external database generalization using kappa as reference. Very recently, however, generalization of the same algorithm was evaluated on two additional external datasets, in this case reporting a combined average performance of κ = 0.61, almost in line with the reference human levels in the corresponding cohort (κ = 0.66) [23], but underperforming with respect to the original values reported in [21] (κ = 0.72–0.77).

Four studies [24,61,62,64] validate their approach over the SHHS database. Comparatively performance of our algorithm (κ = 0.61) falls within the range of the reported results (κ = 0.52–0.73). Two studies [24] and [64] have in addition used the ISRUC dataset as external independent test set, with respective results of κ = 0.63 and κ = 0.57–0.68. Of these two previous studies only the results in [24] are fully comparable to ours (κ = 0.61), as they involve the exact same patient selections in both SHHS and ISRUC. The results in [24] correspond to a previous work of the authors using the exact same datasets as in the present work, but using a different neural network architecture. When comparing the average performance of the method presented in [24] across the full set of external datasets used in both studies, it can be shown that the new architecture proposed in the current study improves the overall generalization capabilities both in the local (κ = 0.60 in [24] vs 0.80 in this work) as well as in the external (κ = 0.50 in [24] vs 0.63 in this work) validation scenarios.

## Discussion

This study has addressed the extensive validation of a deep-learning based solution for the automatic scoring of sleep stages in polysomnographic recordings. Proper handling of the different sources of variability associated with the task has been one of the major traditional problems in the development of automated sleep staging systems. While clinical standard guidelines, such as those contained in the R&K [69] or AASM [1] manuals, aim for a certain level of homogenization in the recording and analysis process, inter-database differences are inevitable in practice. Data variability includes differences in the targeted patient populations, recording methods, or human-related interpretability, see [24] for a detailed discussion. Validation procedures reported in the literature have been so far limited. Performance of the reported methods is often extrapolated using small or non-independent datasets, mostly involving data limited to one particular database. Consequently, the performance is usually bounded to a particular data source, risking overfitting bias. Validation studies usually lack of enough data heterogeneity to allow establishment of valid generalizations. Our experimentation, together with the analysis of the existing literature, has shown the non-triviality of translating the estimated model’s local generalization capabilities in the predictability of independent external datasets. When a system trained with some particular data is presented with similar examples, which are gathered from an external database, performance tends to decrease. This result further motivates the necessity of considering external multi-database prediction as a fundamental mandatory step in the validation of this class of systems. It also suggests a critical revision of the related existing literature in this regard.

In this work we wanted to address this issue and challenge our design by evaluating its performance beyond data from a local database testing set (local generalization validation). For this purpose we have expanded our tests to include a wide selection of previously unseen external databases (external generalization validation). Effectively, by comparing both procedures it is possible to better extrapolate the real generalization performance of the method. For that purpose we have intentionally aimed at selecting databases freely available online in order to enhance reproducibility of the experiments. In total, six independent public databases have been included in this study. To our knowledge this is largest number of datasets to have ever been included on a study of this kind.

On this challenging validation scenario, the deep learning architecture proposed in this work has shown good general performance, as compared with both human and automatic references available throughout the literature. We remit to the respective analyses carried out around data collected in Tables 5 and 6. Still, direct comparison of the results with other works has to be performed with caution. Effectively, even when referencing the same database source, studies might differ on the specific used validation approach, the number of involved recordings, or the particular patient conditions in their respective data selections or training partitions. The specific protocols and subject selection details of each particular study can be found within the referenced publications in Tables 5 and 6. Only the results provided on an earlier study of the authors [24] can be directly compared, as they address the exact same database benchmark. In this regard, the new architecture proposed in the current study outperforms the overall generalization capabilities previously achieved both in the local (κ = 0.60 in [24] vs 0.80 in this work) as well as in the external (κ = 0.50 in [24] vs 0.63 in this work) validation scenarios.

Results from our experimentation have shown that the new CNN+LSTM architecture design introduced in this work translates into considerable improved generalization performance. This improvement has been noticeable on both the local and the external database validation scenarios, and across all the tested configuration variants of the proposed neural network architecture. Experimental data have pointed out as well toward the convenience of adding epoch sequence learning mechanisms using an additional LSTM output block, as with respect to the approach of increasing the length of the input pattern on the CNN-only configuration mode. Moreover, as dimensionality of the CNN input space (4x3000xL) is much bigger than the dimensionality of the LSTM input feature space (50xL), scalability of the solution also improves. Overall, the best performance achieved throughout our experimentation has corresponded to the *CNN_LSTM_5* configuration. No further benefits on increasing the length of the sequence beyond the five epochs have been noticed.

On the other hand, our global results have casted doubt on the convenience of using the proposed optional signal pre-filtering step. This result seems counterintuitive at first sight, as filtering was hypothesized to contribute to the homogenization of input data. Thereby, to cancel out patient and database-specific artifacts unrelated to the relevant neurophysiological activity, which could hinder generalization of the resulting models. However, data have not shown a consistent effect across all the tested datasets. More research is hence needed to fully understand the underlying causes of the high inter-dataset variability when using the proposed filtering pipeline. The same variability, on the other hand, evidences once again the importance of using a sufficiently heterogeneous and independent data sample, from a variety of external sources, to allow the establishment of valid and generalizable conclusions about the performance of an automatic scoring algorithm.

Last but not least, our experimentation has shown that the use of an ensemble of local models leads to better generalization performance in comparison with the use of individual local models alone, hence confirming our preliminary results [24]. In this regard, it is a well-known result that incrementing the amount and heterogeneity of the input training data is an effective approach to achieve better generalization in machine learning. The proposed ensemble approach, however, provides additional advantages in terms of scalability and flexibility of the design. That means the ensemble can be easily expanded by adding new local models when new training data, or new datasets, are available. Moreover, the possibility to develop models based on local datasets reduces the necessity to exchange patient data between different centers, otherwise needed to increase heterogeneity of one big learning dataset. This addresses potential issues in relation with preservation of patient privacy. Altogether, our results thus motivate further exploration of the proposed ensemble-based design in future investigations.

Some possible limitations of our study should be mentioned as well. Specifically, although the proposed ensemble strategy suggests a quantitative improvement in the generalization capabilities among independent databases, there is still notable degradation in the generalization performance in reference to the corresponding local testing datasets. The origin of this degradation must be studied in more detail, investigating alternative approaches to possibly reduce these differences. On the other hand, analysis of the literature regarding human inter-scorer variability has suggested that differences between local and external validation scenarios are likely to affect human experts in a similar manner. As the goal for an automatic scoring algorithm (in which the reference gold standard is based on subjective human scorings) is to achieve comparable agreement with respect to the human inter-scorer levels, it remains to be investigated how much of this degradation can actually be explained by the same intrinsic effect in human scoring. For this purpose, the reference levels of expected human agreement, and the corresponding local-external validation differences, need to be assessed for each particular database subject to validation. However, among the databases used in this study, reference levels of human scoring variability were only available for the HMC, SHHS, and ISRUC datasets, all of them constrained to a local validation scenario. Further investigation is therefore needed involving databases for which the reference levels of human agreement are available including the external validation scenario.

In the case of the SHHS dataset a random selection of 100 PSG recordings was performed, however we could have used more data available for this cohort and study the generalization effects of the derived local models. Some recent studies have suggested that diversity of data plays a more important role on generalization than the amount of data itself [64], however our study did not include a specific protocol to test this hypothesis. Future work might explore increasing the local sample size and add additional datasets to the testing benchmark. Future research will also include the exploration of alternative ensemble combination strategies. The Naive-Bayes combiner [70], for example, might be an appealing approach in taking advantage of the different output probability distributions associated with each individual model in the ensemble. Better hyper-parameterization and data pre-processing methods must be also investigated. In particular, variability of the results for the Dublin dataset with respect to the proposed filtering pipeline remain unclear, and need to be studied in more detail. Finally, future work will be conducted toward addressing the effects of the input sampling rate (in this study signals were resampled to 100 Hz) and study the contribution of the selected input signal derivations to the resulting model’s generalization capabilities.

## Supporting information

S1 Table Summary characteristics of the datasets included in the experimentation. (DOCX)

S1 File List of randomly selected recordings integrating the SHHS dataset. (TXT)
