# Supplementary material for: Inter-database validation of a deep learning approach for automatic sleep scoring
Source: PLoS One. 2021 Aug 16;16(8):e0256111. doi: 10.1371/journal.pone.0256111 (PMC8366993; doi:10.1371/journal.pone.0256111)
Supplement: S1 Table — (DOCX) [file pone.0256111.s001.docx]

**S1 Table. Summary characteristics of the datasets included in the experimentation**

| **Dataset** | **Source** | **Acquisition device** | **Used derivations** | **Sampling Rate (in Hz)** | **Pre-filtering**  **(in Hz)** | **Mains frequency (in Hz)** | **Physical range** |
| --- | --- | --- | --- | --- | --- | --- | --- |
| HMC | Sleep Center, HMC Haaglanden, The Netherlands | SOMNOscreen Plus and 10-20 (SOMNOmedics, Germany) | EEG1 C3-M2  EEG2 C4-M1  EOG E2-E1  EMG submental (bipolar)  ECG modified lead-II | 256  256  256  256  256 | 0.2 – 35/128  0.2 – 35/128  0.2 – 35/128  0.2 – N/A  0.2 – N/A | 50 | ±800 μV  ±800 μV  ±800 μV  ±800 μV  ±2400 μV |
| Dublin | Sleep Disorders Clinic at St Vincent's University Hospital, Dublin | Jaeger-Toennies system (Erich Jaeger GmbH, Germany) | EEG1 C3-A2  EEG2 C4-A1  EOG Left-Right  EMG submental  ECG modified lead-II | 128  128  64  64  128 | 0.30 – 35  0.30 – 35  0.30 – 35  10 – 75  0.30 - 75 | 50 | — NV (Normalized Volts) |
| SHHS | Sleep Health Heart Study (SHHS) multi-center cohort, USA | Compumedics P-series Sleep Monitoring system, versions 3-4, (Compumedics Limited, Australia) | EEG1 C3-A2  EEG2 C4-A1  EOG Left-Right  EMG submental (bipolar)  ECG modified lead-II | 125/128  125/128  50/64  125/128  250/256 | 0.15 – N/A  0.15 – N/A  0.15 – N/A  0.15 – N/A  0.15 – N/A | 60 | ±125 μV  ±125 μV  ±125 μV  ±31.5 μV  ±1.25 mV |
| Telemetry | Leiden University Hospital, The Netherlands | Telemetry system (Kemp et al. [1], The Netherlands) | EEG1 Pz-Oz  EEG2 Fpz-Cz  EOG horizontal  EMG submental  N/A (no ECG recorded) | 100  100  100  100  N/A | 0.03 – 800  0.03 – 800  0.03 – 800  0.03 – 800  N/A | 50 | ±3000 μV  ±3000 μV  ±3000 μV  ±3000 μV  N/A |
| DREAMS | University of MONS - TCTS Laboratory, and Université Libre de Bruxelles - CHU de Charleroi Sleep Laboratory, Belgium | Brainnet (MEDATEC, Belgium) | EEG1 Cz-A1  EEG2 Fp1-A2  EOG (P8-P18)  EMG submental  ECG modified lead-II | 200  200  200  200  200 | 0.16 – 70  0.16 – 70  0.16 – 70  10 – 70  0.16 - 70 | 50 | ±800 μV  ±800 μV  ±800 μV  ±800 μV  ±3 mV |
| ISRUC | Sleep Medicine Centre of the Hospital of Coimbra University (CHUC), Portugal | SomnoStar Pro (SensorMedics Corporation, USA) | EEG1 C3-M2  EEG2 C4-M1  EOG E2-E1  EMG submental  ECG | 200  200  200  200  200 | 0.3 – 35*  0.3 – 35*  0.3 – 35*  10 – 70*  N/A  *All Notch filtered 50 Hz as well | 50 | ±25 μV  ±25 μV  ±25 μV  ±101 μV  ±87 μV |

**S1 Table. Summary characteristics of the datasets included in the experimentation (continuation)**

| **Dataset** | **Population characteristics** | **Reference for sleep scoring** | **Number scorers** | **Number instances** | **Dataset class distribution (Proportion of W, N1, N2, N3, R)** |
| --- | --- | --- | --- | --- | --- |
| HMC | Random selection of 154 (88 M, 66 F) PSG recordings from the sleep center database containing a mix of patients affected of different sleep disorders. Selection includes both in-hospital and ambulatory recordings. Age: 53.8 ± 15.4; AHI: 14.6 ± 17.0; ArI: 20.1 ± 15.2 | AASM 2.4 2017 | 10 | 139145 | 0.17, 0.11, 0.36, 0.19, 0.15 |
| Dublin | 25 (21M, 4F) full overnight PSGs from adult subjects with suspected sleep-disordered breathing (possible diagnosis of obstructive sleep apnea, central sleep apnea or primary snoring). Subjects had to be above 18 years of age, with no known cardiac disease, autonomic dysfunction, and not on medication known to interfere with heart rate. Age: 50 ± 10 years, range 28-68 years; BMI: 31.6 ± 4.0 kg/m², range 25.1-42.5 kg/m²; AHI: 24.1 ± 20.3, range 1.7-90.9 | Rechtschaffen and Kales | 1 | 20774 | 0.23, 0.16, 0.34, 0.13, 0.15 |
| SHHS | Random subset of 100 PSG recordings gathered from the Sleep Health Heart Study (SHHS) follow-up 2. Inclusion criteria included age 40 years or older, no history of treatment of sleep apnea, no tracheostomy, and no current home oxygen therapy. Sample does not discard patients with cardiovascular disorders. | Modified Rechtschaffen and Kales (check manual of operations for details [2]) | 2-5 | 108965 | 0.25, 0.04, 0.44, 0.11, 0.16 |
| Telemetry | 44 whole-night PSGs obtained from a study of temazepam effects on sleep in 22 Caucasian males and females without other medication. Subjects had mild difficulty falling asleep but were otherwise healthy. The PSGs of about 9 hours were recorded in the hospital during two nights, one of which was after temazepam intake, and the other of which was after placebo intake | Rechtschaffen and Kales | 8 | 42691 | 0.10, 0.09, 0.47, 0.15, 0.20 |
| DREAMS | 20 whole-night PSG recordings coming from healthy subjects. These recordings were specifically selected for their clarity (i.e. that they contain few artifacts) and come from persons, free of any medication, volunteers in other research projects, conducted in the sleep lab | AASM 2007 | 1 | 20242 | 0.18, 0.07, 0.41, 0.19, 0.15 |
| ISRUC | 100 subjects (55 male, 45 female) with evidence of having sleep disorders (subgroup-I). Most of the subjects have detected sleep apnea events; the subjects could be under medication, but all were in position to breathe without the help of machine. Age 20-85, avg.=51, std.=16 years | AASM 2007 | 2 (scorings from expert 1 are used in this study as reference) | 90187 | 0.23, 0.13, 0.31, 0.19, 0.13 |

**References**

| [1] | B. Kemp, A. Janssen en M. Roessen, „A digital telemetry system for ambulatory sleep recording,” in *Sleep-Wake Research in The Netherlands*, vol. 4, M. Coenen en J. Arends, Red., Dutch Society for Sleep Wake Research, 1993, pp. 129-132. |
| --- | --- |
| [2] | Case Western Reserve University, "Sleep Heart Health Study: Reading center manual of operations," Case Western Reserve University, Cleveland, Ohio, 2002. |
